# Analgesics promote welfare and sustain tumour growth in orthotopic 4T1 and B16 mouse cancer models

**DOI:** 10.1177/0023677217739934

**Published:** 2017-12-05

**Authors:** Jennifer Lofgren, Amy L Miller, Claudia Chui Shan Lee, Carla Bradshaw, Paul Flecknell, Johnny Roughan

**Affiliations:** 1 Unit for Laboratory Animal Medicine, University of Michigan Medical School, Ann Arbor, Michigan, USA; 2 School of Agriculture, Food and Rural Development, Newcastle University, Newcastle upon Tyne, UK; 3 Pain and Animal Welfare Science Group, Institute of Neuroscience, Comparative Biology Centre, Newcastle University, Newcastle upon Tyne, UK

**Keywords:** mouse, buprenorphine, NSAID, meloxicam, analgesia, cancer, refinement

## Abstract

Murine orthotopic cancer models often require surgery, potentially causing pain or distress. However, analgesics are often withheld because they may alter tumour development. Two orthotopically implanted cancers were investigated in mice pre-treated with meloxicam (10 mg/kg), buprenorphine (0.2 mg/kg) or saline (1 ml/kg). Tumours were imaged and welfare was assessed using body weight, behaviour and nociceptive responses. In study 1, BALB/c mice were inoculated with 4T1 mammary carcinoma or saline during surgery or anaesthesia. As pre-treatment with a single buprenorphine dose appeared beneficial to cancer growth consistency, a second cohort of mice additionally received saline or buprenorphine at 12 and 24 h. Surgery resulted in increased mammary tumour growth and lung metastases. These unwanted effects were lessened by buprenorphine pre-treatment, especially when given repeatedly. Mammary tumour-bearing mice became less active and nociceptive thresholds declined over time, indicating some discomfort as tumours grew. In study 2, C57BL/6 mice received B16 melanoma. This non-surgical model was used to determine whether meloxicam or buprenorphine affected cancer seeding of the lungs. While meloxicam reduced B16 lung seeding, buprenorphine did not. Mechanical thresholds decreased as cancer developed in mice bearing melanoma, but the magnitude of this was insufficient to conclude that there were any significant welfare concerns. This study highlights the scientific value in utilising non-surgical models, where possible. When surgery must be performed at the time of tumour inoculation, the effects of this should be controlled with appropriate analgesics to enhance the value and possibly translation of the research.

## Résumé

Les modèles orthotopiques murins de cancer exigent souvent une chirurgie, laquelle peut potentiellement causer douleur et détresse, mais les analgésiques sont souvent exclus car ils peuvent altérer le développement des tumeurs. Deux cancers orthotopiques implantés ont été étudiés chez des souris préalablement traitées par méloxicam (10 mg/kg), buprénorphine (0.2 mg/kg) ou une solution saline (1 ml/kg). Les tumeurs ont été imagées et le bien-être social a été évalué en mesurant le poids du corps, le comportement et les réponses nociceptives. Dans l'étude 1, les souris BALB/c ont été inoculées avec un carcinome mammaire 4T1 ou une solution saline pendant la chirurgie ou l'anesthésie. Le prétraitement avec une dose unique de buprénorphine s’étant révélé bénéfique pour la constance de la croissance du cancer, une deuxième cohorte de souris a reçu en plus une solution saline ou de la buprénorphine à 12 et 24 heures. L'opération a entraîné une augmentation de la croissance des tumeurs mammaires et des métastases pulmonaires. Ces effets indésirables ont été atténués par la buprénorphine utilisée en prétraitement, surtout lorsqu'elle était administrée de façon répétée. Les souris porteuses de tumeurs mammaires sont devenues moins actives et les seuils nociceptifs ont diminué au fil du temps, indiquant la présence d’un certain malaise au fur et à mesure que les tumeurs augmentaient. Dans l'étude 2, des souris C57BL/6 ont été inoculées par un mélanome B16.

Ce modèle non-chirurgical a été utilisé pour déterminer si l'ibuprofène ou la buprénorphine affectaient l'ensemencement des poumons par le cancer. Bien que le méloxicam ait réduit l'ensemencement des poumons par le mélanome B16, la buprénorphine n'a pas eu cet effet. Les seuils mécaniques ont diminué au fur et à mesure que le cancer s'est développé chez les souris porteuses du mélanome, mais l'ampleur de cette diminution s’est avérée insuffisante pour conclure à l'existence de préoccupations en matière de bien-être. Cette étude souligne la valeur scientifique de l'utilisation de modèles non-chirurgicaux, dans la mesure du possible. Lorsque la chirurgie doit être effectuée au moment de l'inoculation de la tumeur, ses effets devraient être contrôlés par des analgésiques afin d’accroître la valeur et peut-être la translation de la recherche.

## Abstract (de)

Orthotopische Maus-Krebs-Modelle bedürfen oft chirurgischer Eingriffe und verursachen potenziell Schmerz oder Leid. Analgetika werden jedoch häufig nicht verabreicht, da sie u. U. die Tumorentwicklung verändern können. Zwei orthotopisch implantierte Karzinome wurden bei Mäusen untersucht, die zuvor mit Meloxicam (10 mg/kg), Buprenorphin (0.2 mg/kg) oder Salzlösung (1 ml/kg) behandelt worden waren. Tumore wurden bildlich dargestellt und das Wohlbefinden wurde anhand von Körpergewicht, Verhalten und nozizeptiven Reaktionen bewertet. In Studie 1 wurden BALB/c Mäuse während der Operation oder Anästhesie mit 4T1 Mammakarzinom oder Salzlösung inokuliert. Da die Vorbehandlung mit einer Einzeldosis Buprenorphin einem gleichbleibenden Krebswachstum förderlich schien, erhielt eine zweite Mäusekohorte zusätzlich Salzlösung oder Buprenorphin nach 12 und 24 Stunden. Die Operation resultierte in erhöhtem Mammatumorwachstum und Lungenmetastasen. Diese unerwünschten Folgen wurden durch Vorbehandlung mit Buprenorphin verringert, insbesondere bei wiederholter Verabreichung. Mammatumor tragende Mäuse wurden inaktiver und nozizeptive Schwellen sanken mit der Zeit als Anzeichen für gewisses Unbehagen angesichts zunehmenden Tumorwachstums. In Studie 2 erhielten C57BL/6-Mäuse B16-Melanom. Dieses nicht-chirurgische Modell wurde verwendet, um zu ermitteln, ob Meloxicam oder Buprenorphin die Krebsbesiedlung der Lunge beeinflussten. Meloxicam reduzierte die B16-Lungenbesiedlung, Buprenorphin jedoch nicht. Mechanische Schwellen sanken mit zunehmender Karzinomentwicklung bei Melanom tragenden Mäusen, jedoch in einem Ausmaß, das unzureichend war, um schlussfolgern zu können, dass signifikante Beeinträchtigungen des Wohlbefindens zu befürchten sind. Diese Studie unterstreicht den wissenschaftlichen Wert des Einsatzes nicht-chirurgischer Modelle, soweit möglich. Wenn zur Tumorinokulation eine Operation erfolgen muss, sollten die Auswirkungen mit entsprechenden Analgetika kontrolliert werden, um den Wert und möglicherweise die Überleitung der Forschungsergebnisse zu optimieren.

## Resumen

Los modelos murinos con cáncer ortotópico a menudo requieren cirugía, lo que suele provocar dolor o malestar. No obstante, los analgésicos a menudo son negados ya que pueden alternar el desarrollo del tumor. Se investigaron dos cánceres implantados ortotópicamente en ratones que habían recibido meloxicam (10 mg/kg), buprenorfirna (0.2 mg/kg) o salina (1 ml/kg). Los tumores fueron escaneados y se analizó el bienestar utilizando el peso corporal, el comportamiento y las respuestas nociceptivas. En el estudio 1, los ratones BAB/c fueron incoculados con carcinoma de mamas 4T1 o salina durante cirugía o anestesia. Ya que un tratamiento previo con una única dosis de buprenorfina pareció ser beneficioso para la consistencia de crecimiento del cáncer, un segundo cohorte de ratones también recibió salina o buprenorfina a las 12 y 24 horas. La cirugía provocó un crecimiento del tumor de mama y una metástasis pulmonar. Estos efectos no deseados fueron aliviados con un tratamiento previo con buprenorfina, especialmente al administrarse repetidamente. Los ratones con tumor de mama fueron menos activos y los límites nociceptivos decrecieron con el tiempo, lo cual indicaba un malestar a medida que los tumores crecían. En el estudio 2, los ratones C57BL/6 recibieron melanoma B16. Este modelo no quirúrgico fue utilizado para determinar si el meloxicam o la buprenorfina afectaban la germinación del cáncer en los pulmones. Mientras que el meloxicam redujo la germinación pulmonar B16, la buprenorfina no lo hizo. Los límites mecánicos decrecieron a medida que el cáncer se desarrollaba en los ratones con melanomas, pero la magnitud de esto fue insuficiente para concluir que había una preocupación significativa por el bienestar de los animales. Este estudio destaca el valor científico que tiene utilizar modelos no quirúrgicos, siempre que sea posible. Cuando se debe realizar intervenciones quirúrgicas en el momento de la inoculación del tumor, los efectos deberían controlarse con analgésicos adecuados para aumentar el valor y la posible implementación del estudio.

Large numbers of mice are involved in cancer research. Many receive orthotopic tumour inoculation, whereby tumours grow in the tissue of origin. This approach is supposed to maximise the translational relevance of results,^1,2^ whereas with heterotopic tumour inoculation tumours grow in an unrelated tissue, for example subcutaneously. The tumour cells implanted may be from different strains of mice or even different species. They can be grown in immunocompromised mice, or as in this case, they can be syngeneic derived tumour cell lines. Although tumour inoculation can be a minimally invasive procedure, some procedures such as intra-ocular injection may not be, and some models require surgery, for example laparotomy to implant hepatocellular carcinoma. Although this could be painful, analgesics are often withheld due to concerns about their potentially confounding effects on tumour development. A search for the terms ‘mouse AND tumour AND surgery’ on https://www.ncbi.nlm.nih.gov resulted in 49,266 articles. When combined with the terms ‘analgesia’, ‘opioid OR buprenorphine’, or ‘nonsteroidal anti-inflammatory drug (NSAID) OR NSAID OR meloxicam OR carprofen OR ketoprofen OR indomethacin OR acetaminophen OR paracetamol’, that number decreased to 818 articles: a difference of 48,448 articles concerning studies with both tumours and surgery in mice that listed no analgesics. It may be that many of these actually used analgesia but did not disclose it.^3^ However, such under-reporting is not usually the case.^4^ Of those studies that did list analgesics, most evaluated their effects on tumour-associated pain or therapeutic effects on tumour growth. For example, whereas NSAIDS have been found to impede tumour development in some orthotopic tumour models in rodents,^5–7^ some opioids can enhance cancer growth, often via suppression of the immune system.^8,9^ However, pain or stress due to surgery, anaesthesia or even sub-optimal housing can also alter tumourigenesis.^10–16^ Analgesics including buprenorphine and indomethacin can minimise surgery-associated changes, bringing tumour development closer to that observed in a non-surgical model. The mechanism could involve aspects such as preventing hypothalamic–pituitary–adrenal (HPA) axis up-regulation or minimising immunological impacts, such as maintaining natural killer cell functioning.^8,17^

The present investigations aimed to evaluate the impacts of meloxicam or buprenorphine on tumour growth in two orthotopic mouse cancer models: in BALB/c mice inoculated with 4T1 mammary carcinoma, and then in C57BL/6NCrl mice with B16 melanoma. The 4T1 work also assessed the effects of using surgery for tumour implantation, which rather unusually is optional, and of using single- or multiple-buprenorphine dosing. It was hypothesised that provision of pain relief, rather than being confounding, might both improve welfare and enhance study validity.

## Materials and methods

### Ethical approval

All work was undertaken according to the Animals (Scientific Procedures) Act 1986 under Home Office Licence Authority with approval from the Newcastle University Animal Welfare Ethical Review Body (AWERB).

### Design

**Figure 1.**
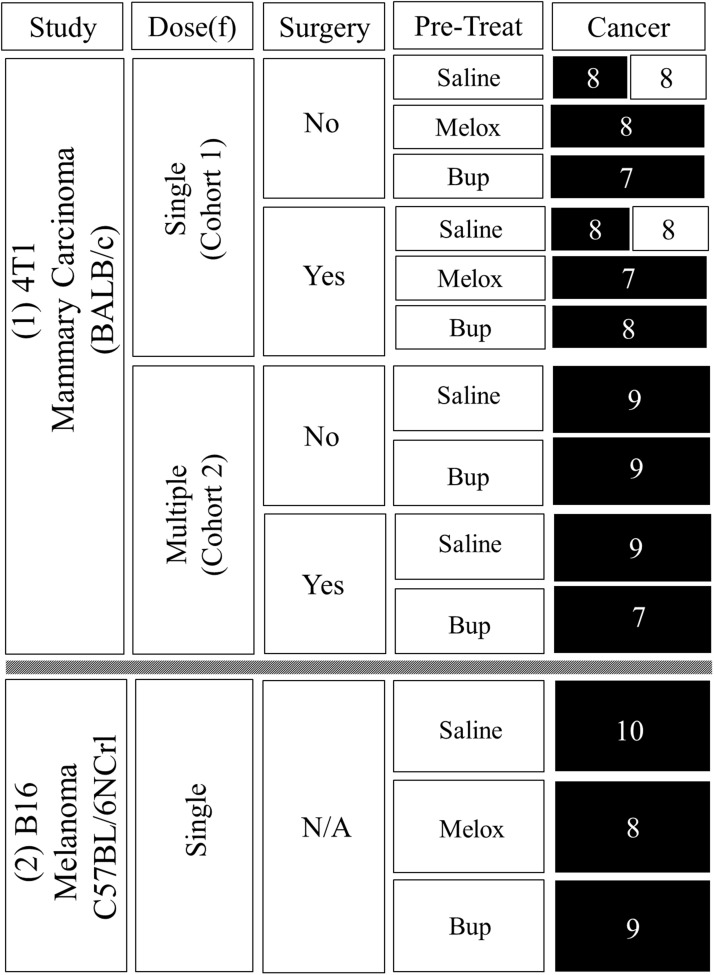
Treatments combinations and final numbers. Mice were female BALB/c inoculated with saline or 4T1 mammary carcinoma (Study 1), or female C57BL/6NCrl inoculated with B16 melanoma (Study 2). 4T1 mice underwent laparotomy or only anaesthesia (Surgery = Yes/No). Cohort 1 received one (Dose(f) = Single) s/c pre-inoculation injection (Pre-Treat) of saline (1 ml/kg), meloxicam (Melox: 10 mg/kg) or buprenorphine (Bup: 0.2 mg/kg). Cohort 2 received the same saline or buprenorphine pre-treatment as cohort 1, but treatment was repeated at 12 and 24 h (Dose(f) = Multiple). Boxes in the ‘Cancer’ column contain mouse numbers. Black boxes show groups inoculated with cancer and clear boxes those that were non-cancer controls. Mice in the B16 study (Study 2) did not have surgery (surgery = N/A). All were inoculated with B16 melanoma after one s/c pre-treatment of either saline (1 ml/kg), meloxicam (10 mg/kg) or buprenorphine (0.2 mg/kg).

Figure 1 shows the final mouse numbers and treatments. Individuals were assigned using a random number generator (https://www.random.org). As concerns about using analgesics in cancer studies mainly relate to tumour growth alterations, the intention was to investigate from a ‘severe case’ perspective, so the analgesic dose rates were purposefully high compared with those usually recommended.^18^

**Figure 2.**
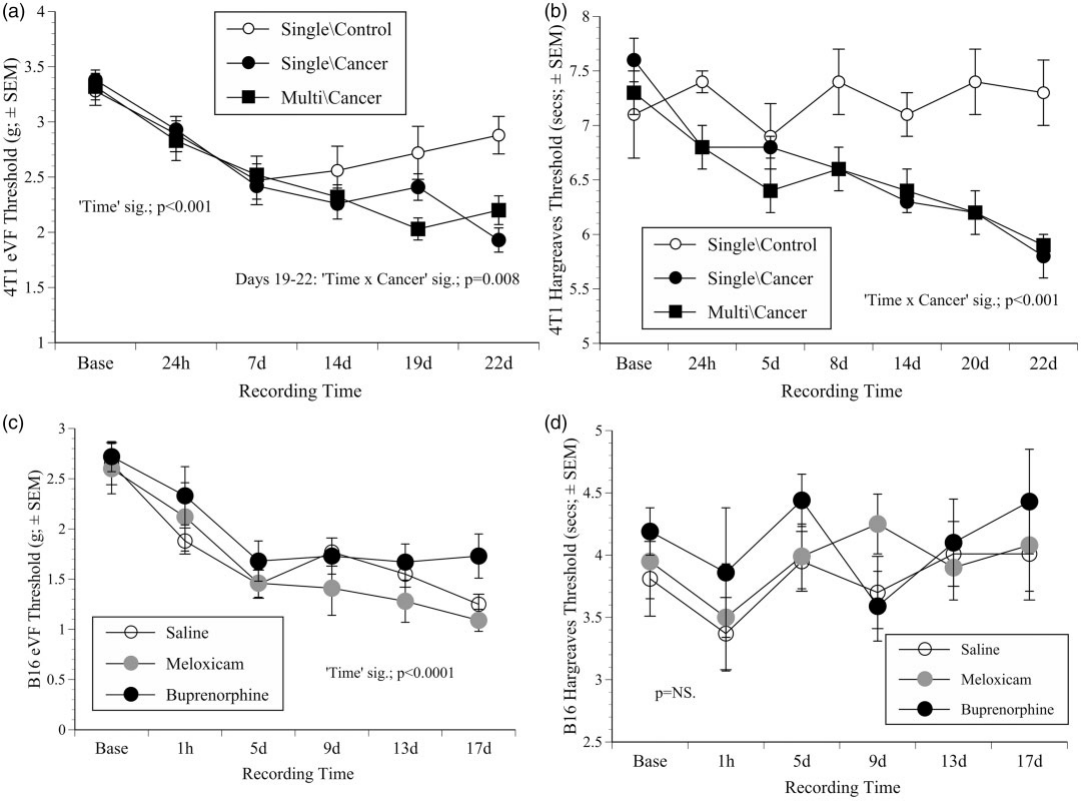
Nociceptive testing results. (a) Mean (combined paws) electronic von Frey (eVF) thresholds (grams ± 1SEM) in BALB/c mice in the single- and multiple-dose 4T1 cohorts versus non-cancer controls; showing the significant decline over time in all mice, but more so in cancer groups. (b) Left paw thermal withdrawal thresholds (mean ± SEM) in the same groups as (a); showing increased thermal sensitivity as tumours grew. (c) eVF threholds (both paws) in the B16 groups where thresholds generally declined during tumour development. (d) Mean thermal (both paws) withdrawal thresholds (±SEM) in the B16 groups showing thermal response thresholds were not significantly altered as cancer developed. Significant (Sig.) ‘factors’ from repeated measures ANOVA and *p*-values are indicated.

Welfare was assessed using body weight changes, peripheral nociceptive sensitivity alterations (using von Frey and Hargreaves testing) and by recording behaviour changes post-procedurally and as cancer developed. All mice were initially pre-treated with a single dose of meloxicam, buprenorphine or saline prior to 4T1 or B16 cancer inoculation. The 4T1 mammary carcinoma cell line was tested as cells are inoculated orthotopically; however, this can be achieved either non-surgically or surgically, via percutaneous injection, or following surgical exposure of the fat pad.^19^ We wished to assess whether, as has previously been suggested,^19^ unnatural surgical implantation actually provides a more reliable model of human breast cancer, and evaluate the impact of distress or post-surgical pain on tumour development. Study 1 therefore had tumours implanted both in conjunction with surgery or using anaesthesia only. Mice were inoculated with 4T1 mammary carcinoma or saline, the latter to permit assessment of the effects of surgery or anaesthesia in the absence of analgesics or cancer. Having assessed the effects of a single buprenorphine dose, study 1 was extended to examine multiple doses. Accordingly, a second cohort of mice was tested, with additional buprenorphine or saline being given at 12 and 24 h following tumour inoculation. Initial analyses found no indication that it would be beneficial to test multiple doses of meloxicam.

The two cohorts in study 1 are respectively referred to as the 4T1 single-dose and multiple-dose groups. All mice in cohort 2 received 4T1 cancer inoculation.

Studies of B16 melanoma do not usually involve surgery. Cells are injected intravenously and primarily seed the lungs. Therefore, in study 2 all mice were inoculated intravenously with B16 melanoma following a single pre-treatment with saline, meloxicam or buprenorphine. The aim was to determine whether meloxicam or buprenorphine altered metastatic tumour spread to the lungs.

Group sizes were established using data from a previous study where between 8 and 10 mice per group achieved 80 to 85% power.^20^

### Animals and husbandry

Mice were supplied by Charles River (Margate, Kent, UK). They were certified free of the common pathogens listed on their website (http://www.criver.com/products-services/basic-research/health-reports/europe-asia/uk-by-species). They weighed 18 ±0.25 g. For study 1 (4T1 mammary carcinoma) 108 female BALB/c mice were used. Study 2 used 30 female C57BL/6NCrl mice inoculated with B16 melanoma. These strains were chosen because they are syngeneic for the tumour cell line each was to be inoculated with. The 4T1 cells used in study 1 were derived from spontaneous mammary tumours in BALB/c mice, whereas the B16 melanoma cells used in study 2 were from C57BL/6 mice with spontaneously developing melanoma. They were acclimatised for one week in groups of 2–3 in individually ventilated cages (Arrowmight, Hereford, UK) containing hardwood bedding (Aspen, BS and S Ltd, Edinburgh, UK), a cardboard tube, chew blocks and sizzle nest (B and K Universal, Hull, UK). Food (R&M no.3, SDS Ltd, Essex, UK) and tap water was provided *ad libitum*. Cages were cleaned once per week. The holding room was kept at 22 ±2℃ and 27–40% humidity on a 0700–1900 light cycle.

### Tumour inoculation and treatments

The tumour cells were obtained from Caliper Life Sciences (Waltham, MA, USA) and were cultured following vendor recommendations. They were confirmed pathogen free by the IMPACT Profile I (PCR) at the University of Missouri Research Animal Diagnostic and Investigative Laboratory. The cells were luciferase-expressing 4T1-luc2 mammary carcinoma (4T1) (developed by Kim et al.^21^) or B16-F10-luc-G5 melanoma (B16) (Xenogen Corporation, Alameda, California, USA). All inoculations were performed between 8 and 11 a.m. in batches of 8–10 mice.

In the 4T1 study mice were anesthetised with isoflurane in oxygen (4–5%; 0.5–1 l/min) and maintained at 2–3% isoflurane in oxygen (0.25–0.5 l/min) via a nose cone. Body temperature was kept between 36℃ and 38℃ using a heat pad (Harvard Apparatus, Edenbridge, Kent, UK). Absence of pedal withdrawal responses was used to ensure adequate depth. After eye ointment was applied (Pliva Pharma Ltd., Zagreb, HR) the mice were subcutaneously (s/c) injected with saline (1 ml/kg), buprenorphine (0.2 mg/kg; ‘Vetergesic’, Reckitt-Coleman, Hull, UK) or meloxicam (10 mg/kg; Boehringer Ingelheim, Labiana Life Sciences S.A., Terrassa, Spain). The fourth mammary gland was then inoculated percutaneously with 100 μl Dulbecco’s Phosphate Buffered Saline (DPBS) containing 1 × 10^6^ 4T1-luc2 cells, or the same volume of 0.9% sterile saline.

Mice in the multiple-dose 4T1 study (cohort 2) were subcutaneously treated with saline (1 ml/kg) or buprenorphine (0.2 mg/kg) before tumour inoculation, and then at 12 and 24 h after tumour inoculation. All mice in the 4T1 study were prepared as if for surgery; the abdomen was first shaved and sprayed with chlorhexidine (Hydrex derma-spray, Leeds, UK), but only half subsequently underwent a 1.5 cm midline laparotomy lasting ∼15 min. This was completed exactly as previously described,^22^ but without abrasion of the ileum, and the skin was closed using a continuous subcuticular suture (4/0 polydioxanone; Ethicon, Livingston, UK) rather than mattress sutures. The same veterinary surgeon performed all surgeries. The non-surgery mice underwent 15 min of isoflurane anaesthesia only.

In the B16 study mice received a single s/c pre-treatment with saline (1 ml/kg), meloxicam (5 mg/kg) or buprenorphine (0.2 mg/kg) just prior to restraint in a 50 ml syringe cartridge modified to expose the tail. They then received an intravenous (i/v) injection of 100 μl of DPBS containing 5 × 10^5^ B16-F10-luc-G5 melanoma cells via the tail.

All mice recovered in a warming cabinet set at 35 ±1℃ for 30 minutes before being returned to the animal holding room. Apart from when mice were imaged, all data were collected in the holding room by treatment-blinded staff. All staff involved in data collecting data were cross-trained to ensure consistency.

### Nociceptive testing

Mechanical response thresholds were obtained using ‘The Mousemet’ electronic von Frey (eVF) device (Topcat Metrology, Ely, UK). Animals were placed into one of four separate raised runs that had Plexiglas sides and steel rod floors. After a 10 min acclimation period six readings were obtained from each hind-paw, allowing at least 2 min between each. The probe force rise rate was 1 g/s applied when animals were stationary. The 4T1 eVF thresholds were tested at baseline (between 12 and 4 p.m. the day before inoculation), at 24 h following recovery from anaesthesia, and then on days 7, 14, 19 and 22. In the B16 study eVF readings were taken at baseline, 1 h and on days 5, 9, 13 and 17. Equipment malfunction meant baseline eVF readings were missed in the first 10 4T1 mice.

Thermal nociceptive testing used a Hargreaves apparatus (Model 37370; Ugo Basile, Italy). This had six clear plastiglas enclosures (11 cm × 17 cm × 14 cm) to which mice were first acclimated for 5 min. Baseline latencies were obtained between 12 and 4 p.m. the day before tumour inoculation. In the 4T1 study Hargreaves data were subsequently recorded at 24 h, and on post-inoculation days 5, 8, 14, 20 and 22. The B16 recordings were at baseline, 1 h and on days 5, 9, 13 and 17. Heat intensity was 280 mW/cm^2^ with a 30 s cut-off time. Three readings were obtained from each hind-paw, allowing ≥2 minutes between each, again when mice were stationary.

### Behaviour recordings

Mice were filmed individually for 10 min in three clear cages containing only sawdust (Type 1144B, Techniplast UK Ltd, Northamptonshire, UK). Recordings were made between 1 and 4 p.m. using three Canon Legria HFM 506 cameras placed 30 cm from each cage. The 4T1 recordings were one day before inoculation (baseline), at 3 and 24 h, and then on days 5, 8, 14, 20 and 22. The B16 mice were filmed at baseline, 1 h and on days 5, 9, 13 and 17.

### Imaging

D-Luciferin (PerkinElmer, Beaconsfield, UK) was dissolved in DPBS to 15 mg/ml and frozen at −80℃. Mice were anaesthetised with isoflurane in batches of three and imaged in an IVIS Spectrum 200 (PerkinElmer, Beaconsfield, UK) on a stage maintained at 36℃. Anaesthesia was provided by face-mask delivery of 1.5–2% isoflurane in 0.5 l/min oxygen. Once thawed to room temperature, 150 mg/kg luciferin was injected s/c. Peak reactivity occurred 12 min later, so after this open filter scans were taken at 7, 12, 15, 19 and 22 days in the 4T1 study. The B16 mice were imaged at 24 h and on days 5, 9, 13, 17 and 19.

### End-point criteria/terminal assessment

Mice were weighed daily and examined according to United Kingdom Coordinating Committee for Cancer Research (UKCCCR) guidelines.^23^ The end-point was reached if there was >20% body weight loss alongside poor coat condition and mobility. If these signs were present, after a final IVIS scan mice were to be euthanased by cervical dislocation without recovery from anaesthesia. Any remaining B16 or 4T1 mice were to be euthanased on days 19 or 22 respectively. All mice underwent a necropsy with *ex-vivo* imaging of primary tumours, kidney, liver, intestines and lungs. The 4T1 primary tumours were removed and measured with calipers (Mitutoyo, UK Ltd). In the 4T1 study 12 mice had to be excluded due to tumour inoculation error, which resulted in the rapid development of carcinomatosis. There were no correlations between these occurrences and treatment or surgery. Three B16 mice were also excluded, two due to injection error, and one that developed a very large ovarian metastasis. Apart from these no other mice were euthanased early and the exclusions left sufficient final numbers (shown in Figure 1).

### Data analysis

**Figure 3.**
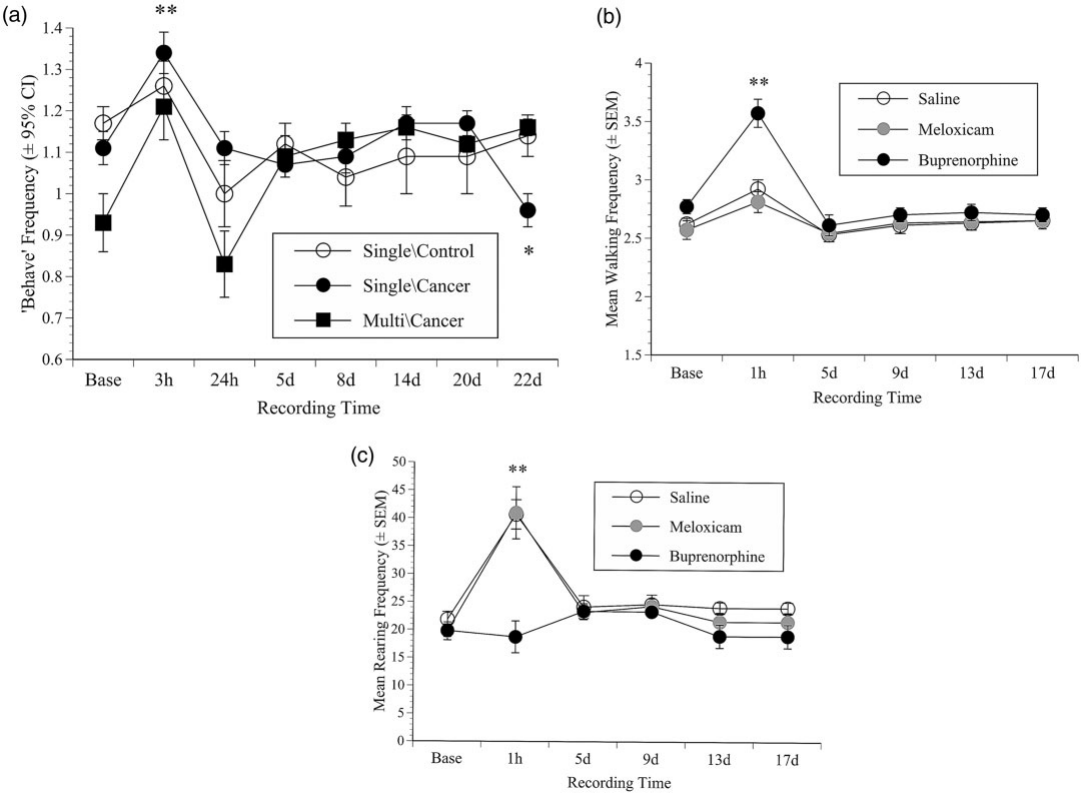
Behaviour results. (a) The geometric mean frequency (±1SEM) of the summary measure ‘Behave’ (+95% Confidence Intervals (CI)) in mice in the 4T1 single- or multiple-dose cohorts versus non-cancer single-dose controls, illustrating the post-procedural activity enhancement but relatively normal behaviour, even when cancer was well developed. The mean frequency (±SEM) of walking (b) or rearing (c) in the B16 groups; illustrating that at 1 h mice walked more following s/c saline (1 ml/kg) or meloxicam (10 mg/kg), but especially more after buprenorphine (0.2 mg/kg) compared with baseline (**: *p* < 0.001(b)). All mice also reared more at 1 h (**: *p* < 0.001(c)) except those that received buprenorphine. The effects of buprenorphine and those in the meloxicam and saline groups subsided by five days and behaviour was subsequently normal.

**Figure 4.**
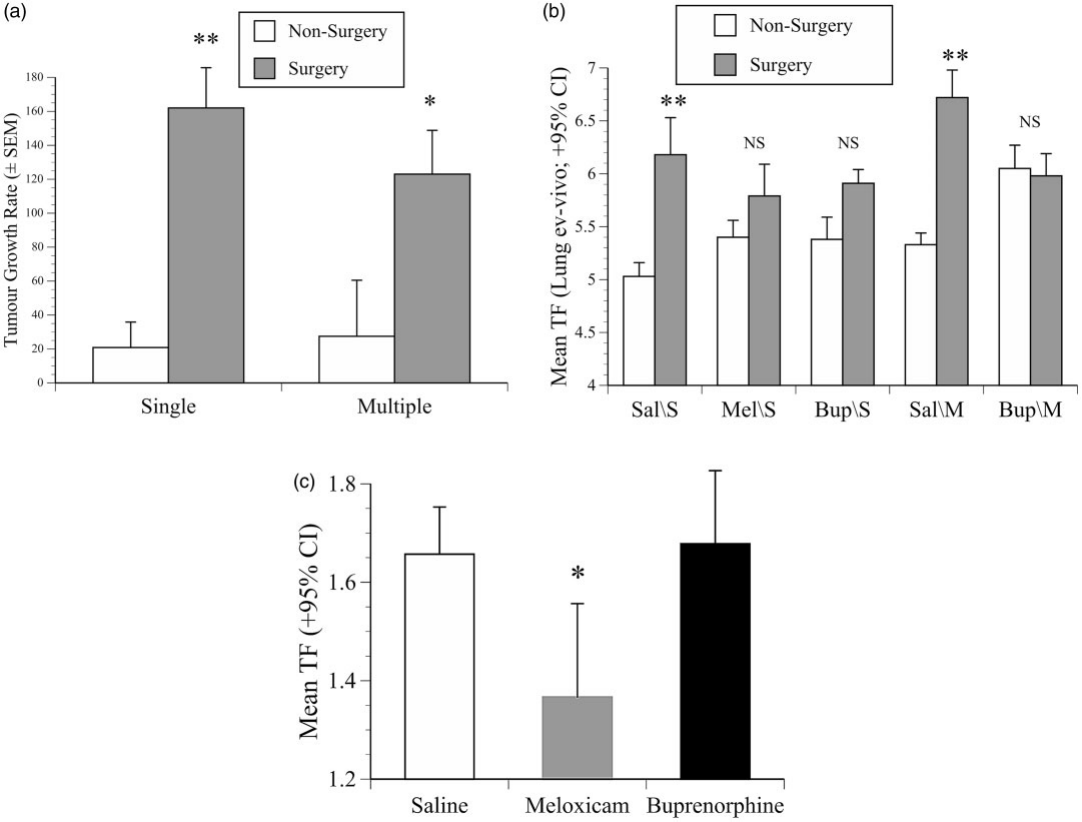
Imaging results. (a) Mean rate of development of signals from 4T1 primary tumours over days 7–22 (TF ×10^9^ photons/s/cm^2^/day ±SEM) in mice that underwent surgery or not, illustrating the significant greater surgery-related tumour growth rate in both 4T1 cohorts (**: *p* < 0.001: *: *p* < 0.05). (b) Geometric mean TF (photons/s/cm^2^±95% CI) of *ex-vivo* metastatic lung signals in mice in the single (S) or multiple (M) dose 4T1 cohorts pre-treated with saline (Sal; 1 ml/kg), meloxicam (Mel; 10 mg/kg) or buprenorphine (Bup; 0.2 mg/kg) before undergoing surgery or not. In the multiple- and single-dose saline groups (Sal/S or Sal/M) surgery significantly increased lung metastases (**: *p* < 0.001), but buprenorphine given singly and especially repeatedly (Bup/S, Bup/M) protected against greater tumour spread (buprenorphine-treated surgery vs. non-surgery groups, *p* = NS). (c) The geometric mean TF (photons/s/cm^2^±95% CI) on the last day in the B16 study, illustrating meloxicam pre-treatment significantly reduced lung seeding compared with saline or buprenorphine (*: *p* < 0.05).

All statistical analyses were performed using the Statistics Package for the Social Sciences (SPSS software version 22.0, SPSS Inc., Chicago, USA). The two cohorts in the 4T1 study had to be analysed separately. Repeated measures ANOVA with probability corrected multiple comparisons (Bonferroni) was used. In the 4T1 study the between-subjects factors were ‘drug’, ‘surgery’ and ‘cancer’ status, and ‘time’ was the within-subjects factor. The B16 analysis included the factors ‘drug’ and ‘time’ only. The baseline eVF thresholds were highly uniform, so to balance the analysis the missing 4T1 values were replaced with the corresponding group mean. The 4T1 eVF data showed no baseline left or right paw bias so values were averaged at each time-point. The 4T1 Hargreaves data showed a clear paw bias so these data were analysed separately. Automated behaviour analysis software (HomeCageScan (HCS): Version 3; Clever Systems Inc., USA) was used to obtain the frequency of rearing and walking (‘Rear-up’, ‘Come Down’, ‘Remain Rear-up’ and ‘Walk Left’, ‘Right’ or ‘Slow’) from the video material at each time-point. The 4T1 behaviour data required Log_10_ transformation before undergoing ANOVA. Figure 3(a) therefore shows back-transformed means with ±95% confidence intervals (±95%CI). In the B16 study the raw behaviour data were acceptable. Living Image software (PerkinElmer, Beaconsfield, UK) quantified tumour burden as the Total Flux (TF; photons/sec/µW/cm^2^) of bioluminescent signals within auto-generated (2% threshold) regions of interest (ROIs) over the mammary or lung regions, with additional ROIs over the abdomen to detect any other metastases. TF on the last day provided a measure of 4T1 final burden; however, as signals emerged by day seven it was also possible to estimate the average growth rate as the change in TF from day seven to the final scan on day 22 ((TF on day 22 – TF on day 7)/15). No signals were apparent in the B16 study until day 17, which was two days before the final scan, so only the final TF values were assessed. The 4T1 *ex-vivo* primary tumour burden (volume) was calculated from the caliper measurements (4/3*II*r^3^). Bivariate correlation analysis (Pearson’s *R*) was used to determine how well the imaging data predicted true tumour burden. Apart from the 4T1 growth data (Figure 4(a)) all imaging data, including in the B16 study were Log_10_ transformed before applying ANOVA. Figures 4(b) and 4(c) therefore show back-transformed means +95%CI. All other results are mean values ±1SEM. The B16 weight data underwent ANOVA as total change from baseline. The 4T1 bodyweight data were non-homogeneous so percentage weight changes were used. Original data are available from an online repository (http://figshare.com).

## Results

### 4T1 study

#### Body weight: Surgery and multiple doses of buprenorphine was associated with loss in body weight

Six mice were excluded, having lost between 11 and 24% of baseline weight by day one (>3 times the group average). They were all in the cohort 1, one from each drug-treated non-surgery cancer group, two from the saline non-cancer surgery group and two cancer-treated mice that received meloxicam before surgery. This reduced group sizes to six or seven, which was considered acceptable. Over the first three days the single-dose surgery groups lost weight whereas the non-surgery mice gained, but only −2 versus +2% (*f*(1, 52) = 9.1, *p* = 0.004). Neither cancer inoculation nor a single dose of meloxicam or buprenorphine resulted in significant weight change. Mice in the multiple-dose cohort lost only negligible weight following surgery. Multiple-dose buprenorphine was associated with 2% greater weight loss over the first three days (*f*(1, 31) = 5.5, *p* = 0.02). This was more obvious in the non-surgery group, but was not significant. By day six all mice exceeded baseline weight and showed continued gains until day 19, with no lasting effects of surgery or pre-treatment. Although cancer-bearing mice in each cohort lost weight as the study ended, this was not significantly more than non-cancer controls.

#### Nociceptive testing: Multiple doses of buprenorphine significantly increased mechanical thresholds, whereas cancer growth significantly decreased them

All groups showed comparable baseline eVF thresholds. Mice given buprenorphine repeatedly in conjunction with surgery (all of which were cancer inoculated) were the only group that showed significantly increased eVF thresholds (greater force to respond) (*f*(3, 28) = 7.5, *p* = 0.001), that is, decreased nociception, but only at the 24 h time-point. As the other mice responded similarly in the days following treatment the responses were pooled within the single- or multiple-dose cancer-bearing groups (ignoring surgery) and the two saline, non-cancer control groups. Figures 2(a) (eVF) and 2(b) (Hargreaves) show the results. The eVF thresholds declined more over time in cancer-bearing mice in both the single- and multiple-dose cohorts (‘Time’ (*f*(5, 49) = 8.4, *p* < 0.001; *f*(5, 26) = 21.5, *p* < 0.001, respectively), but most obviously spanning days 19–22 in mice in the single-dose cohort (Figure 2(a); ‘Time’ × ‘Cancer’ interaction (*f*(5, 49) = 3.5, *p* = 0.008)), indicating increased nociception as the tumours grew.

#### Cancer growth reduced the latency to withdraw from a noxious heat source

Hargreaves latencies were also unaffected by surgery or pre-treatment. Responses from each paw generally occurred sooner over time, but the left paw responded sooner than the right. As a result, Figure 2(b) shows data from this paw only with groups combined as in Figure 2(a). Mice in the single-dose cohort showed a modest latency decrease from baseline to 24 h (*f*(1, 53) = 5.5, *p* = 0.023), indicating decreased nociception. After this cancer development was associated with a further reduction in response latency (‘Time’ × ‘Cancer’ interaction; *f*(6, 318) = 5.6, *p* < 0.001), that is, tumour growth was associated with increased nociception. In the multiple-dose cohort both the left and right paws were sensitised by 24 h (right: *f*(1, 31) = 7.3, *p* = 0.001; left: *f*(1.31) = 18.2, *p* = 0.0001), and both paws then responded sooner as tumours grew (right: *f*(6,186) = 2.1, *p* = 0.049; left: *f*(6, 186) = 5.3, *p* < 0.0001), but as the significance values indicated, the left paw became the most sensitised.

#### Behaviour: While surgery and analgesic treatments did not significantly alter behaviour, spontaneous active behaviours decreased as tumours grew

Rearing and walking were unaffected by drug administration or surgery at any point during either the single- and multiple-dose 4T1 studies. As a result, these behaviours were combined to create a summary variable ‘Behave’ which was used to assess any later effects of cancer development in each cohort of mice.^22,24^ Figure 3(a) shows the activity changes in the control groups (saline, non-cancer) versus the cancer-bearing mice in the single- and multiple-dose cohorts. At 3 h all mice were more active than at baseline (*f*(1, 61) = 15.8, *p* = 0.001; *f*(1, 34) = 9.1, *p* = 0.005, cohorts 1 and 2, respectively). This dissipated over 24 h and activity was relatively normal until between days 20 and 22, where compared with non-cancer controls, tumour-bearing mice in cohort 1 showed a greater activity decline (‘Time’ × ‘Cancer’ interaction; *f*(1, 61) = 7.0, *p* = 0.011). However, this was not apparent in the equivalent cancer-bearing mice in cohort 2.

#### Tumour growth as indicated by IVIS signal intensity: Surgery was a more significant tumour growth modulator than analgesia

Signal intensity increased more rapidly in mice in cohort 1 that underwent surgery compared with non-surgery controls (20.8 ± 15^9^ vs. 162 ± 24 × 10^9^ photons/s/day; *f*(1, 38) = 24.8, *p* < 0.0001, Figure 4(a)), and they also showed stronger signals on the last day (*f*(1, 38) = 25.8, *p* < 0.001). In cohort 2 the surgery/non-surgery growth difference was somewhat less, but still significantly greater in the mice that underwent surgery (27.5^9^ ±33 vs. 123 ±26 × 10^9^ photons/s/day; *f*(1, 31) = 5.1, *p* = 0.03). The *ex-vivo* caliper measurements indicated larger volume tumours in mice that underwent surgery (554 ±50 vs. 420 ±40mm^3^; all cancer-bearing groups combined). The lung scanning results reiterated the impact of surgery, where in both cohorts the surgery groups showed more extensive lung seeding (single: *f*(1, 44) = 12.1, *p* = 0.001; multiple: *f*(1, 35) = 9, *p* = 0.005, Figure 4(b)). The greatest spread was in the groups pre-treated with saline before surgery either as a single dose (Sal/S; *f*(1,16) = 9.7, *p* < 0.001) or given repeatedly (Sal/M; *f*(1,19) = 22.4, *p* < 0.001). Notably, metastatic signals in the meloxicam or buprenorphine groups in cohort 1 (Mel/S, Bup/S) were similar to those in the non-surgery groups (*p* = 0.258, *p* = 0.23, respectively), and in cohort 2 the surgery/non-surgery groups given buprenorphine repeatedly were almost identical (Bup/M; *p* = 0.83). The *ex-vivo* measurements showed mice in cohort 1 with the largest tumours had more metastases (Pearson’s *r* = 0.34, *p* = 0.024) and more rapid tumour growth over days 7–22 (*R*^2 ^= 0.4, *p* < 0.001).

### B16 study

#### Body weight: Mice treated with buprenorphine lost slightly more weight than those receiving saline or meloxicam

The three test groups had similar initial weights (22.8–23.6 g) and there were no significant losses on day one. By day three mice receiving buprenorphine lost more weight than those given saline or meloxicam (*f*(1,27) = 3.8, *p* = 0.036), but an average of only 1 g more. After this the saline and meloxicam groups maintained weight and the buprenorphine group gained, but only slightly more (∼1 g). Nevertheless, there was a significant ‘Time’ effect and a ‘Time’ × ‘Pre-treatment’ interaction (*f*(17, 408) = 5.8, *p* < 0.001; *f*(17, 408) = 2.7, *p* < 0.001, respectively). There were no significant weight changes as the study ended.

#### Nociceptive testing: Mechanical thresholds, but not latency to withdraw from thermal stimulus, significantly decreased as cancer developed

Baseline eVF thresholds were similar across the three groups and both paws responded similarly (2.7 ±0.6 g; Figure 2(c)). Pre-treatment had no effect on post-inoculation thresholds which continued to decline as cancer developed (‘Time’ significant; *f*(5, 120) = 30, *p* < 0.0001). Although there was a partial stabilisation between days five and nine, thresholds were lower than at baseline at every subsequent time-point (*p* ≤ 0.001). The baseline B16 Hargreaves latencies were also similar initially, and although they also declined from baseline to 1 h (from 3.9 ±0.7 to 3.5 ±1.1 s; Figure 2(d)), not significantly (*p* = 0.06).

#### Behaviour: Buprenorphine significantly increased walking behaviour

Figures 3(b) and 3(c) illustrate rearing and walking frequency separately because they were affected by buprenorphine differently. Overall, both were significantly increased at 1 h following tumour inoculation (rearing: *f*(1, 26) = 151, *p* < 0.001; walking: *f*(1, 26) = 58, *p* < 0.001); however, relative to the behaviour of mice in the meloxicam or saline groups, at the 1 h time-point pre-treatment with buprenorphine was associated with increased walking (Figure 3(b); *f*(2, 26) = 24.6, *p* < 0.001) and less rearing (Figure 3(c); *f*(2, 26) = 13.5, *p* < 0.001). Both rearing and walking became normal by day five and remained as such until the study ended.

#### Tumour growth, as indicated by IVIS signal intensity: Meloxicam, but not buprenorphine, reduced lung metastases

There was no significant change in TF until day 17, but after this development occurred rapidly. There was no difference in final day signal intensity between mice that had received saline or buprenorphine, but as Figure 4(c) shows, signal intensity was lower in mice that received meloxicam (*p* = 0.014, *p* = 0.01; meloxicam vs. saline or buprenorphine, respectively). Neither drug affected abdominal metastases.

## Discussion

Out of concern that analgesics can alter tumour development, animal cancer studies often withhold them. However, this ignores the possibility that cancer-related pain or distress could cause unnecessary scientific variation. Two studies were undertaken to assess whether buprenorphine or meloxicam altered tumour growth in BALB/c mice developing 4T1 mammary carcinoma, and in C57BL/6NCrl mice inoculated with B16 melanoma. Both studies used luciferase-expressing cells lines so tumour growth and spread could be determined using bioluminescent imaging. The 4T1 study included groups undergoing tumour inoculation in conjunction with surgery. A secondary aim was to assess any pain the inoculation procedures might cause, and whether pain occurred as tumours grew. This was assessed using body weight and behaviour changes, and by recording parallel signs of nociceptive responding via thermal and mechanical nociceptive threshold testing. It was hypothesised that mice undergoing surgery would show behaviour changes and nociceptive sensitivity changes suggesting underlying pain, but that these effects would be minimised in the groups pre-treated with meloxicam or buprenorphine. Neither the B16 or 4T1 inoculation procedures appeared to cause significant pain, and there was also little evidence of pain as each type of cancer developed. It was predicted that the B16 inoculation and 4T1 percutaneous inoculation procedure would be relatively harmless, but the relatively benign effect of undertaking 4T1 inoculation paired with surgery was unexpected. The main findings were that surgery significantly increased 4T1 tumour growth and metastatic spread, but provision of pre- and post-operative buprenorphine neutralised the impact of surgery on the tumour model. Providing peri-procedural analgesia was therefore shown to be beneficial, by more closely approximating tumour growth to the spontaneous development experienced by human patients that this research is designed to model.

The BALB/c mice in the 4T1 study were predicted to show an impact of surgery comparable with that in another of our recent investigations in which BALB/c mice underwent laparotomy but showed substantially reduced behavioural activity and significant post-surgical weight losses.^22^ However, on this occasion they generally became more active following either anaesthesia or surgery and maintained body weight. Albeit the previous BALB/c mice were male, the literature on sex-related pain sensitivity is highly non-consensual. The most recent knowledge also suggests females should be more, rather than less, prone to pain,^25^ so although it cannot be discounted, sex was thought unlikely to account for such opposing post-surgical outcomes. More relevantly perhaps, the aim of the previous study was to validate use of an agent that was supposed to enable imaging of inflammation. The laparotomy procedure was therefore modified such that after the agent was injected it included 60 s of visceral abrasion. These additional manipulations may have previously been aggravating to normal behaviour, and it was possibly also relevant that the two studies had different surgeons. Whereas the previous study used mattress sutures, in this study a continuous subcuticular suture was used to close the skin. This may have been a refinement that helped to limit alterations in behaviour, but it is impossible to verify whether these protocol variations contributed to the lesser impact of surgery found here. An obvious limitation of this study is that we did not have the opportunity to replicate the study to determine if the pain responses measured were reproducible.

The other noticeable effect of surgery was that both thermal and mechanical sensitivity had increased at 24 h. However, this change was also apparent in 4T1 non-surgery controls, indicating pain alone may not have been the source of this sensitivity. Alterations as a result of drug treatment were evidenced by the increase in 4T1 mechanical thresholds at 24 h following multiple-dose buprenorphine; however, this was only seen following surgery and not, as would have been predicted, in the equivalent non-surgery group. Finally, stress causes analgesia; but it is severe or prolonged the opposite can occur, that is, hyperalgesia.^26–28^ However, while the stress of the surgery may have been sufficient to cause hyperalgesia, the relatively minor procedure used in the B16 investigation was unlikely to be sufficient to induce this level of stress.

The only obvious behavioural change in either study was in the buprenorphine-treated B16 mice, which showed a substantial initial increase in walking and lack of rearing. These were most probably non-specific effects as previously seen in buprenorphine-treated mice following either vasectomy or laparotomy.^24,29^ In general, the activity increases following B16 inoculation may have reflected the normal tendency of mice to explore a novel environment, in this case the filming cage. Although hyper-locomotion can be caused by chronic stress,^30,31^ it is again doubtful this could have been the case following the short period of restraint needed for B16 inoculation.

Nociceptive responding continued to increase as 4T1 tumours grew (Figure 2(a) and 2(b)), and in the B16 study there was a less obvious but still significant reduction in mechanical thresholds as the study ended (Figure 2(c)). The nociceptive threshold was reduced, indicating that the mice experienced greater pain. However, by the time these changes were most obvious, the mice in both studies had been exposed to numerous different potential stressors. Apart from being isolated from cage-mates during nociceptive testing and filming, they underwent repetitive anaesthesia for imaging, daily weighing and a health inspection, and in the 4T1 study also restraint for tumour palpation. It is possible that the combined impact of these experiences was sufficient to cause some degree of hyperalgesia. In a previous study, mice with bladder cancer^20^ demonstrated evidence of pain by showing an enhanced conditioned place preference for morphine as their tumours grew. Here, the B16 mice behaved relatively normally; their mechanical response thresholds showed only a modest decrease as cancer developed, and there were no significant thermal response alterations. As the 4T1 study ended, increased nociceptive sensitivity was accompanied by a minor reduction in behavioural activity confined to mice in cohort 1 and not those bearing the same cancer in cohort 2 (Figure 3(a)).

Imaging was used to determine any drug-related tumour growth changes. Although meloxicam reduced B16 lung seeding as compared with saline or buprenorphine, buprenorphine had no effect on this model of metastatic growth (Figure 4(c)), and neither analgesic had any detrimental impact on 4T1 tumour growth consistency or metastases. However, surgery caused 4T1 tumours to develop more rapidly and spread more (Figure 4(a) and 4(b)). Such changes have previously been attributed to removal of anti-angiogenesis control, growth factor release, or metabolic, neuroendocrine and immunological suppression.^6,11,13^ Several previous studies have also shown stress due to anaesthesia, pain or hypothermia^12,32–34^ can promote many types of cancer.^10–16^ Although it is unknown which aspect contributed most to the present 4T1 acceleration, because it was partially neutralised by buprenorphine, stress again seems the most likely candidate. While surgery alone led to an increase in lung metastases (Figure 4(b)), this was prevented if mice had repeated peri-operative doses of buprenorphine at a dose commonly used for pain prevention.^35^ Previous studies have found analgesics can reduce surgery-associated increases in tumour recurrence,^12,13^ and there are also examples where buprenorphine had either no impact or supported normal tumour growth.^8,9^ Perhaps due to these aspects and its post-surgical stress-preventative properties,^9^ giving buprenorphine was beneficial to model consistency. Although this obviously needs confirmation with other cancer models, it suggests that in some cases, especially in cancer studies requiring surgery, that mice might benefit from receiving some form of pain or stress prevention. Therefore, future research goals include similar evaluations as performed in these studies in additional orthotopic tumour models requiring surgery. Furthermore, a meta-analysis of the relative success of anti-neoplastic therapies developed in mouse tumour models requiring and not requiring surgery, as well as in studies providing or not providing peri-operative analgesia, would allow further exploration of the translation of the results of murine orthotopic tumour models to human health.

Mammary tumours most commonly occur in females and therefore we chose to assess the 4T1 tumours in female mice. However, it might be asked why the B16 mice were also female. Although this has been dealt with before,^20^ it was to answer the need for studies that do not only consider males. A minor point to mention was the good agreement between the caliper measurements and the imaging data. Although manual tumour burden estimates are generally inaccurate, in this case they partially predicted eventual 4T1 tumour spread.

These studies document minimal impacts of analgesia on tumour growth in at least one cancer model. Minimising surgical stress via any means possible seems a logical refinement that could remove some current barriers to precautionary analgesic use in animal cancer studies. While possibly less critical in basic science research, pre-clinical studies intended to directly model benefits for human patients may arguably be best served by treating animals similarly to human cancer patients, where analgesic use is normal and usually beneficial.
